# Effect of extracellular vesicles derived from induced pluripotent stem cells on mesangial cells underwent a model of fibrosis in vitro

**DOI:** 10.1038/s41598-023-42912-6

**Published:** 2023-09-21

**Authors:** Bruno Aristides dos Santos Bronel, Edgar Maquigussa, Mirian Aparecida Boim, Antônio da Silva Novaes

**Affiliations:** Renal Division, Department of Medicine, Universidade Federal de São Paulo, 781 Pedro de Toledo St, 13° Floor, São Paulo, SP 04039-032 Brazil

**Keywords:** Kidney, Molecular biology, Nephrology, Induced pluripotent stem cells, Extracellular signalling molecules

## Abstract

The fibrogenic process plays a significant pathophysiological role in the progression of chronic kidney disease. Inhibition of the renin-angiotensin system (RAS) is one strategy to delay disease progression but does not reverse established fibrosis. In this context, induced pluripotent stem cells (iPSCs) have been considered an alternative due to their regenerative potential. iPSCs exert their effects through paracrine signaling, which releases specific biomolecules into the extracellular environment, either directly or within extracellular vesicle (EVs), that can reach target cells. This study aims to evaluate the potential beneficial effects of iPSC-derived EVs (EV-iPSCs) in an in vitro model of fibrosis using mouse mesangial cells (MMCs) stimulated with TGF-β. EV-iPSCs were obtained by differentially ultracentrifuging iPSCs culture medium. MMCs were stimulated with 5 ng/mL of TGF-β and simultaneously treated with or without EV-iPSCs for 24 h. Markers of inflammation, fibrosis, and RAS components were assessed using RT-PCR, western blotting, and immunofluorescence. Under TGF-β stimulus, MMCs exhibited increased expression of inflammation markers, RAS components, and fibrosis. However, these changes were mitigated in the presence of EV-iPSCs. EV-iPSCs effectively reduced inflammation, RAS activation, and fibrogenesis in this fibrosis model involving mesangial cells, suggesting their potential as a strategy to reduce glomerular sclerosis.

## Introduction

Renal fibrosis is characterized by the excessive accumulation of extracellular matrix (ECM) components, which trigger structural, morphological, and functional alterations in renal cells^1,2^. When subjected to stress or stimulation, renal cells generate pro-inflammatory and profibrotic mediators^2^, further exacerbating fibrosis, and ultimately culminating in the advanced stages of chronic kidney disease.

Mesangial cells are highly susceptible to various damaging stimuli, becoming activated and playing a role in progressive glomerular injury^3^. They possess the capacity to produce and release pro-inflammatory and profibrotic substances, including transforming growth factor beta (TGF-β), which is a crucial mediator of renal fibrogenesis^4^, as well as components of the renin-angiotensin system (RAS)^1,4–6^.

Despite therapeutic advancements such as anti-inflammatory approaches and RAS system inhibition, the progression of glomerular damage and renal function loss is still inadequately controlled^1,7,8^. Consequently, there is a need to explore new approaches to halt the fibrosis process and minimize disease progression^1,9^. Stem cell-based therapy has recently emerged as a promising alternative, with induced pluripotent stem cells (iPSCs) gaining particular attention^10^. iPSCs can be generated by reprogramming adult and differentiated cells^10–12^, acquiring a pluripotent state through retroviral transfection of specific genes into the target cell^10,11^. Despite their potential to differentiate into the three germ layers, the clinical application of iPSCs is limited^10,12^ due to their thrombogenic and oncogenic characteristics^10^. Recent studies have demonstrated that stem cells promote cell repair via a paracrine mechanism facilitated by extracellular vesicles (EVs)^10^. EVs, produced by various cell types, including iPSCs^3,10^, play a crucial role in intercellular communication, regulating physiological and pathological processes in target cells.

EVs encapsulate a diverse range of bioactive molecules, including proteins, DNA fragments, mRNAs, and miRNAs^10,13^, which are synthesized by the parent cells, and can modulate the functionality of recipient cells. Consequently, the composition of EVs is contingent upon the cell type from which they originate and can vary under different physiological or pathophysiological conditions. Notably, iPSC-derived EVs (EV-iPSCs) hold promise as a therapeutic option since they exhibit similar activities to whole iPSCs^10,13–15^, while offering the advantage of reduced side effects^15–18^.

Indeed, EV-iPSCs have been investigated as a potential treatment for various conditions, including hepatological, cardiovascular, and bone diseases. They have demonstrated the ability to elicit anti-apoptotic, anti-inflammatory, antifibrotic, antioxidant, and pro-angiogenic effects^10,13,18–26^. In line with this, the present study aims to assess the impact of EV-iPSCs on an in vitro model of fibrosis utilizing mouse mesangial cells (MMCs) stimulated with TGF-β.

## Results

### Characterization of extracellular vesicles from iPSCs

**Figure 1:**
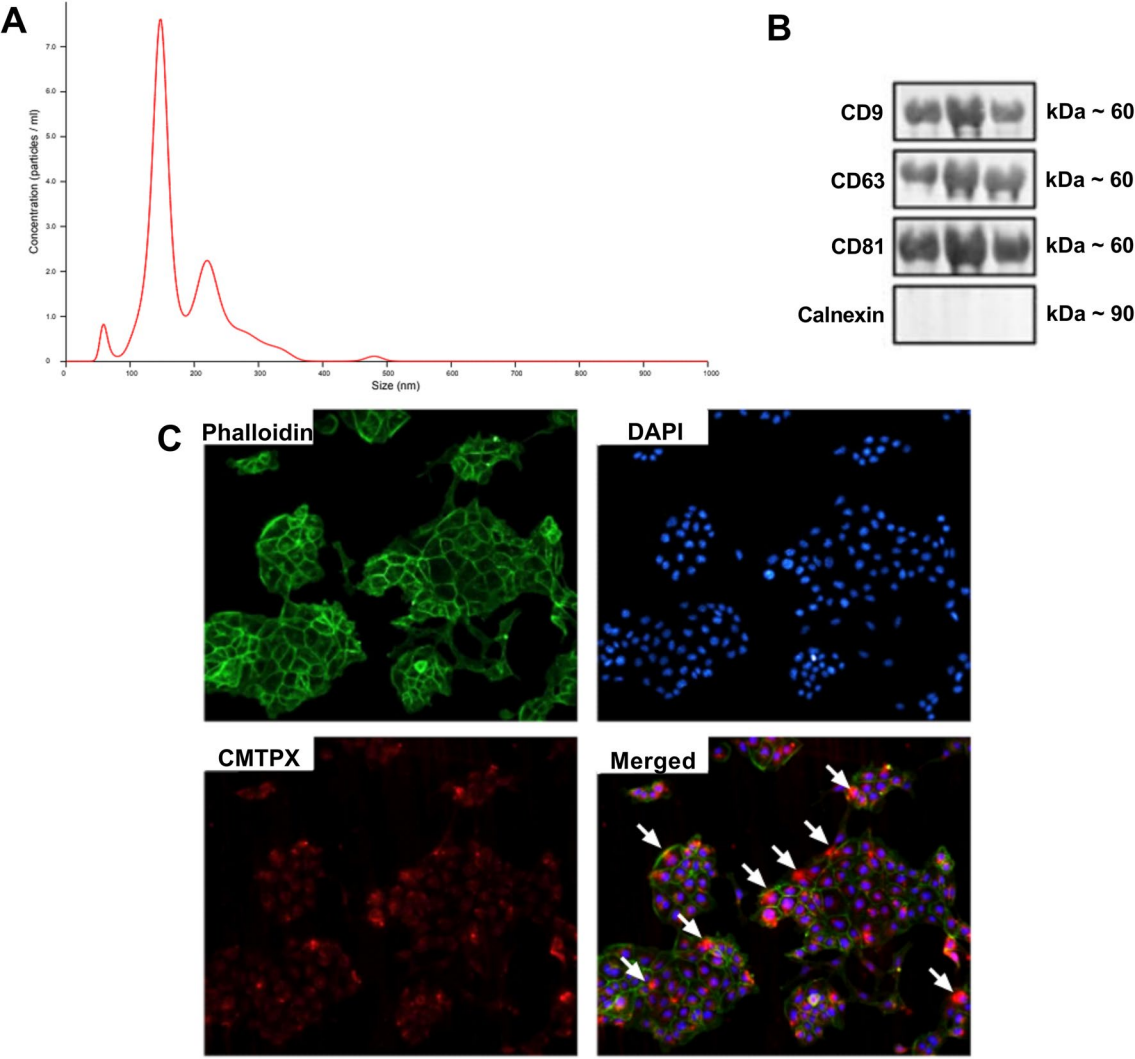
EV-iPSCs characterization and internalization by MMCs. Histogram representing the profile of nanoparticle size by Nanoparticle Tracking Analysis (**A**). Western blot analysis for extracellular vesicle markers (**B**). Cytoskeleton is stained with phalloidin (green) (**C**). The nucleus is stained with DAPI (blue) (**C**). EV-iPSCs are stained with CMTPX (red) (**C**). Colocalization of all markers (**C**). The arrows show the incorporation of EV-iPSCs by MMCs. 20× magnification. Images are representative.

The size and concentration of the EV-iPSCs are depicted in Fig. 1A and Supplementary Fig. 1. The EVs exhibited an average size of 151.6 nm, indicating a prevalence of smaller EVs. The average concentration of EVs was 1.03 × 10^9^ particles/mL, and the presence of EV markers (CD9, CD63, CD81) was confirmed. However, the endoplasmic reticulum marker, calnexin, was not detected (Fig. 1B). These findings suggest that iPSCs generate and release EVs.

### EV-iPSCs were internalized by MMCs

EV-iPSCs stained with CMTPX were observed within the cytoplasm of MMCs, indicating the internalization of EV-iPSCs by these cells (Fig. 1C). The incorporation of EV-iPSCs by MMCs is highlighted by the arrows (Fig. 1C).

### Effect of EV-iPSCs on gene and protein expression of renal fibrosis markers

**Figure 2:**
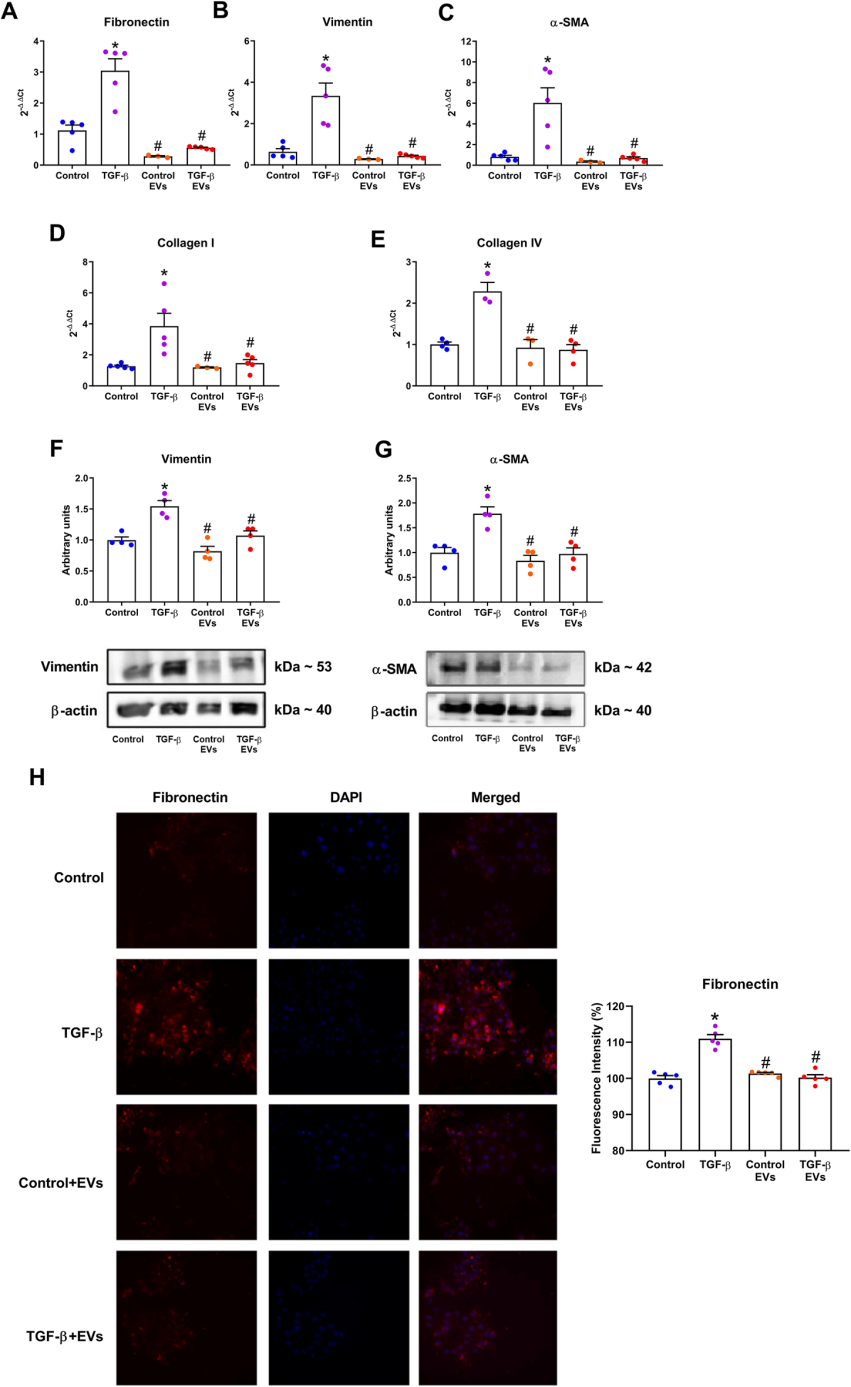
Effect of EV-iPSCs on gene and protein expression of ECM components. The gene expression of fibronectin (**A**), vimentin (**B**), α-SMA (**C**), collagen I (**D**) and collagen IV (**E**) was evaluated by RT-PCR. The protein expression of vimentin (**F**) and α-SMA (**G**) was evaluated by western blotting. HPRT and β-actin were used as the reference gene and protein, respectively. The immunofluorescence of fibronectin was evaluated and quantified by microscopy (**H**). The fibronectin marker is observed in red, and the nucleus was stained with DAPI (blue). 40× magnification. Images are representative of independent experiments. The results are expressed as the mean ± SEM. *p* < 0.05: * versus group control; # versus group TGF-β.

Stimulation of MMCs with TGF-β resulted in elevated gene expression levels of fibronectin, vimentin, α-SMA, collagen I and collagen IV (Fig. 2A–E). Furthermore, TGF-β stimulation significantly increased the protein expression of vimentin and α-SMA (Fig. 2F and G). Immunofluorescence analysis corroborated these findings, demonstrating a significant increase in the protein expression of fibronectin upon TGF-β stimulation (Fig. 2H). However, treatment with EV-iPSCs prevented this response, and no discernible differences were observed between the EV-iPSCs-treated control and TGF-β groups (Control + EVs and TGF-β + EVs), as well as the untreated control group. These results highlighted the ability of EV-iPSCs to modulate the expression of ECM components in target cells, suggesting the potential utility of this strategy in mitigating fibrogenesis mechanisms in TGF-β stimulated mesangial cells.

### Effect of EV-iPSCs on gene and protein expression of RAS components

**Figure 3:**
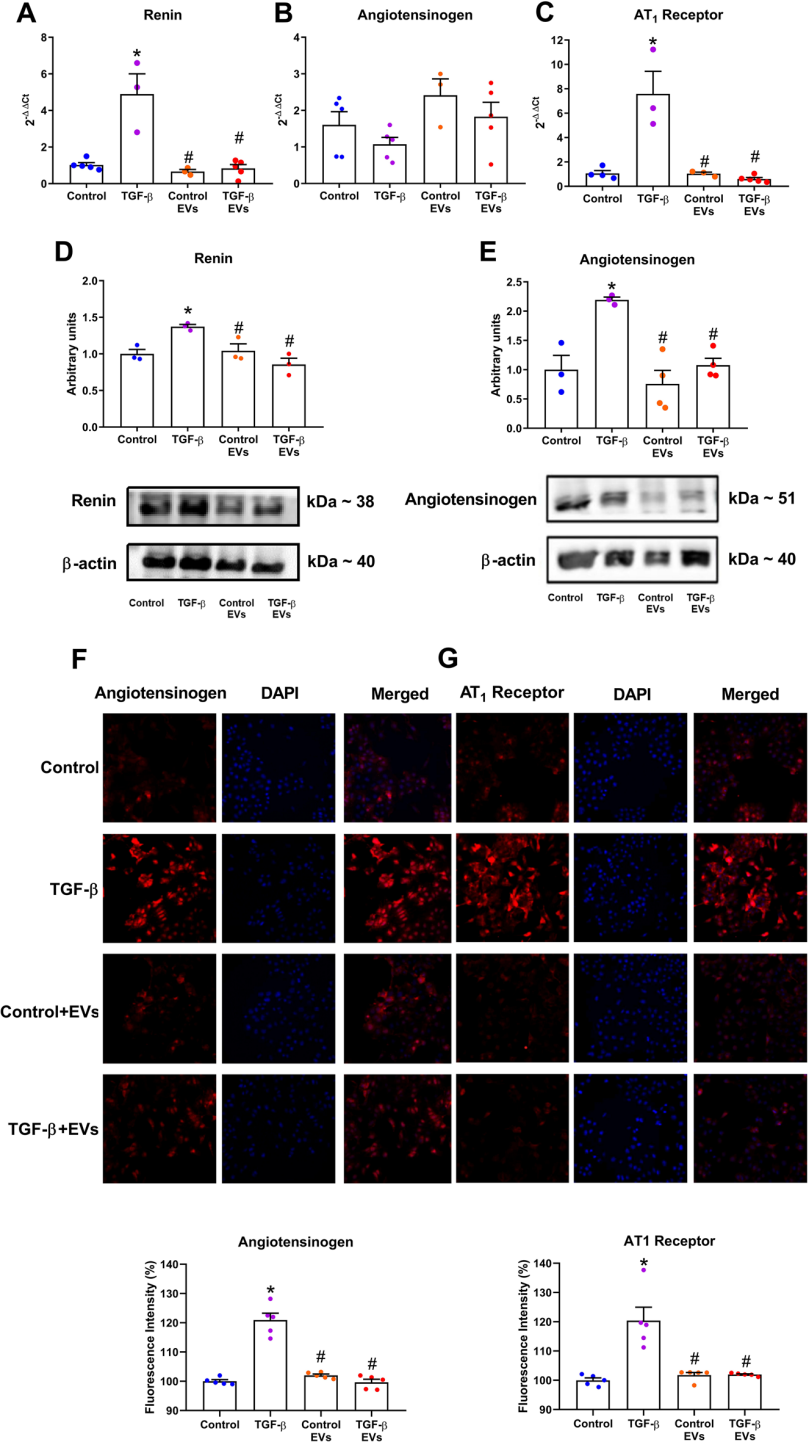
Effect of EV-iPSCs on gene and protein expression of RAS components. The mRNA expression of renin (**A**), angiotensinogen (**B**), and AT_1_ receptor (**C**) was evaluated by RT-PCR. The protein expression of renin (**D**) and angiotensinogen (**E**) was evaluated by western blot. HPRT and β-actin were used as the reference gene and protein, respectively. The immunofluorescence of angiotensinogen and AT_1_ receptor was evaluated and quantified by microscopy (**F**–**G**). Angiotensinogen and AT_1_ receptor markers are observed in red, and the nucleus was stained with DAPI (blue). 40× magnification. Images are representative of independent experiments. The results are expressed as the mean ± SEM. *p* < 0.05: * versus group control; # versus group TGF-β.

MMCs possess the capability to synthesize all components of the RAS, and upon TGF-β stimulation, there was an upregulation of the RAS components involved in RAS activity modulation, namely angiotensinogen, renin and AT_1_ receptor, at both the mRNA and protein levels (as demonstrated by western blot and immunofluorescence) in Fig. 3. Renin and AT_1_ receptors exhibited elevated levels, both mRNA (Fig. 3A and C) and protein (Fig. 3D and G), whereas angiotensinogen levels were increased solely at the protein level (Fig. 3B, E, and F). Notably, the treatment of MMCs with EV-iPSCs prevented all these changes in the gene/protein expression of RAS components, indicating that EV-iPSCs can potentially impede the hyperactivation of RAS induced by TGF-β in mesangial cells.

### Effect of EV-iPSCs on NF-κB-mediated inflammatory and SMAD pathway

**Figure 4:**
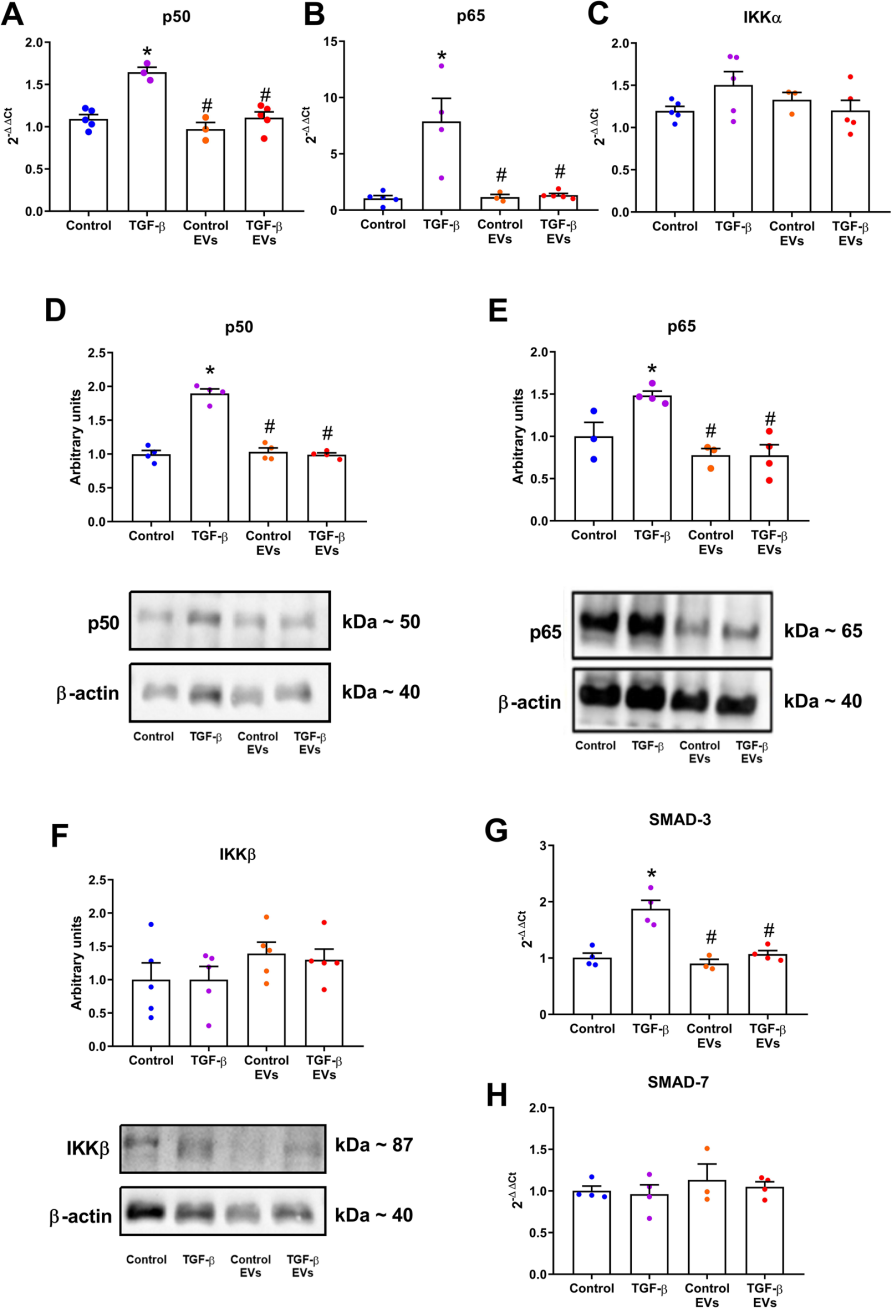
Effect of EV-iPSCs on gene and protein of NF-κB-mediated inflammatory and SMAD pathway. The gene expression of p50 (**A**), p65 (**B**), IKKα (**C**), SMAD-3 (**G**) and SMAD-7 (**H**) was evaluated by RT-PCR. The protein expression of p50 (**D**), p65 (**E**), and IKKβ (**F**) was evaluated by western blot. HPRT and β-actin were used as the reference gene and protein, respectively. The results are expressed as the mean ± SEM. *p* < 0.05: * versus group control; # versus group TGF-β.

To evaluate the activation of components involved in the NF-κB-mediated pro-inflammatory and SMAD pathway, the gene and protein expressions of IKK, p50 and p65 subunits, and gene expression of SMAD-3 and SMAD-7 were examined (Fig. 4). Following TGF-β stimulation, there was a significant increase in both mRNA and protein expressions of the p50 and p65 subunits (Fig. 4A, B, D, and E), while the mRNA and protein levels of IKKα and IKKβ remained unchanged (Fig. 4C and F, respectively) in MMCs. Furthermore, the stimulation with TGF-β resulted in upregulation of SMAD-3 gene expression but did not alter SMAD-7 (Fig. 4G and H). However, treatment with EV-iPSCs prevented the upregulation of these markers. Thus, the administration of EV-iPSCs demonstrated the capacity to modulate the expression of SMAD-3 and NF-κB components, thereby impeding the progression of pro-inflammatory molecules.

## Discussion

The use of EV-iPSCs has shown promising results in terms of tissue function recovery in various organs, including the liver, heart, and brain^21,27,28^. Furthermore, investigating these therapeutic EVs could also prove beneficial for treating kidney diseases^27,28^. In this study, an in vitro model was utilized to assess the impact of EV-iPSCs on MMCs that were stimulated with TGF-β, an inducer of fibrogenesis mechanisms.

The approximate size obtained was 151.6 nm, indicating a predominance of smaller EVs in the sample. These findings align with the minimal information requirements for EVs studies as recommended by MISEV2018^29^, and are consistent with existing literature^24,25,27,28,30–34^, thereby confirming the ability of iPSCs to generate and release EVs. Additionally, using iPSC-derived small EVs, such as the exosomes, has demonstrated more significant therapeutic effects and greater similarity to whole cells, thereby reducing potential side effects^25,27^.

MMCs demonstrated the capability to internalize EV-iPSCs present in the culture medium, suggesting that the delivery of EV contents has the potential to modify gene expression and consequently alter the function of the target cells. This discovery is supported by previous studies conducted by other research groups that investigated the uptake of EV-iPSC by various cell types, including mesenchymal stem cells^32^, endothelial cells^32,33^, cardiomyocytes^35^, liver cells^36^, and renal proximal tubular epithelial cells^31^.

In contrast, it was intriguing to note that EV-iPSCs did not induce any changes in the expression of the assessed molecules in non-stimulated control MMCs. Although visually, it appears to have a statistical difference in gene expression of angiotensinogen between these two groups, the p-value was not significant (Control vs Control + EVs: *p* = 0.1030). This observation suggests that EV-iPSCs do not interfere with the normal functioning of cells. Consequently, it can be inferred that EV-iPSCs do not have any detrimental effects on control cells.

Moreover, this study employed human-derived EV-iPSCs in MMCs. Similar findings have been reported in other studies that utilized human EV-iPSCs in in vivo models. Interestingly, despite the vesicles originating from cells of a different species, the beneficial effects were still observed, suggesting that the molecules delivered by the vesicles likely interacted with mechanisms that are highly conserved across these species, being this a great advantage to use EVs instead of whole stem cells. However, the specific mechanisms involved were not investigated in this study. Furthermore, as demonstrated in previous studies^21–23,25,26,30^, human EV-iPSCs did not elicit immune responses in animal models^23,26^, being suitable for allotransplantation or xenotransplantation. Accordingly, our data showed benefits of using EVs derived from human iPSCs on mouse mesangial cells with no signs of immune response.

As previously established, mesangial cells exhibit high sensitivity to various stimuli, leading to diverse responses such as contraction, production and secretion of vasoactive substances, inflammatory mediators, and profibrotic molecules, among others. In this study, an in vitro model of MMCs was employed to investigate the effects of TGF-β stimulation on inducing inflammatory and profibrotic responses. Consistent with expectations, TGF-β-stimulated MMCs exhibited increased expression of ECM components, and activation of the RAS and NF-κB pathways.

Simultaneous stimulation of MMCs with TGF-β and EV-iPSCs resulted in a reduction of proteins associated with ECM formation. Similar findings were reported by Povero D (2019), who observed a decrease in the profibrogenic phenotype of hepatic stellate cells when treated with EV-iPSCs in vitro and stimulated with TGF-β^36^. Additionally, in vivo studies have demonstrated regenerative following the administration of EVs derived from iPSCs. In a liver fibrosis model, treatment with these EVs led to reduced gene and protein expression of ECM components in mice^36^. Adamiak et al.^27^ also observed that EV-iPSCs reduced interstitial fibrosis in the myocardium using a coronary ischemia and reperfusion model. These findings collectively suggest that EV-iPSCs have the potential to exert an antifibrotic effect, by modulating the gene and protein expression of crucial ECM components.

RAS is a multifunctional system that plays a vital physiological role in both the entire organism and mesangial cells. However, excessive activation of RAS, triggered by various stimuli, including TGF-β, can damage organs and cells. The activation of RAS, as evidenced by the synthesis of its components, contributes to fibrogenesis in glomerular mesangial cells^1,8^. In this study, stimulation with TGF-β in cultured MMCs resulted in increased expression of RAS components and ECM markers. However, treatment with EV-iPSCs inhibited the overexpression of RAS components, indicating that these EVs may modulate RAS activity by regulating the expression of key molecules within this system.

The TGF-β pathway and RAS activation contribute to inflammation in renal tissue through various signaling pathways^4^. SMAD-3, a signaling molecule of the TGF-β pathway, acts as an intermediate in the activation of the NF-κB pathway, demonstrating a fibrotic and inflammatory role in kidney injury^37^. In our study, we observed an upregulation of the p50 and p65 subunits and SMAD-3 in TGF-β-stimulated MMCs, which was reversed by treatment with EV-iPSCs. This finding aligns with existing literature and suggests an anti-inflammatory property of EV-iPSCs^21,28,30^. Notably, neither TGF-β nor EV-iPSCs treatment affected the expression of SMAD-7, an inhibitory SMAD^37^, and IKKα, a molecule that forms a complex with IKKβ to maintain the p50-p65 dimer inactive^38^. Although the present study did not investigate whether EV-iPSCs interfere with the phosphorylation-induced degradation of IκB, leading to NF-κB activation and the phosphorylation of SMADs proteins, this mechanism should not be ruled out and warrants further investigation.

Our findings suggest that EV-iPSCs can modulate gene and protein expression, which may be one of the potential mechanisms underlying the observed beneficial effects in TGF-β-stimulated MMCs. While the specific mechanisms through which EVs influence gene and protein expressions were not assessed in this study, there is evidence supporting the involvement of microRNAs (miRNAs) in this response. Recent studies by Bobis-Wozowicz et al*.* and Povero et al*.* have demonstrated the presence of several miRNAs within EV-iPSCs^36,39^. Notably, miRNA-548c, miRNA-196b, and miRNA-206 exhibit antifibrotic properties^40–42^, while miRNA-1914 can modulate components of the RAS system while exerting an antifibrotic effect^43^. Additionally, miRNA-502 and miRNA-548c possess anti-inflammatory effects^44,45^. Consequently, delivering these miRNAs via EV-iPSCs could potentially modulate the antifibrotic, anti-inflammatory, and RAS pathways. Furthermore, miRNAs may also play a role in the gene expression of angiotensinogen, although further studies are necessary to explore this relationship.

The current study provided evidence of the beneficial properties of EV-iPSCs in TGF-β-stimulated mesangial cells in vitro. One potential mechanism underlying this protective effect is the ability of EV-iPSCs to modulate the expression of genes and proteins involved in profibrotic and pro-inflammatory pathways.

## Methods

### Human induced pluripotent stem cell culture

Human induced pluripotent stem cells (iPSCs) were purchased from ATCC (BYS0112) and cultured at 37 °C in 25 cm^2^ flasks using CellMatrix Basement Membrane Gel (ATCC ACS-3035) in Pluripotent Stem Cell SFM XF (ATCC ACS-3002) medium. To enhance the survival rate of iPSCs, as recommended by ATCC, cells were treated with the ROCK inhibitor Y27632 (ATCC ACS-3030) on the first day after thawing. The flasks were maintained in a humidified environment with 95% air atmosphere and 5% CO_2_. The culture medium was changed daily to prevent cell differentiation. iPSCs were cultured from passage 0 (P0) to P2, and when reaching a confluence of 70–80%, the culture medium was changed. After 24 h, the medium was collected and subjected to differential ultracentrifugation.

### Extracellular vesicles extraction

Differential centrifugation was employed to extract extracellular vesicles from the iPSCs, referred to as EV-iPSCs, present in the culture medium^29^. In summary, the culture medium was collected and subjected to sequential centrifugation steps: first, at 300 g for 10 min, then at 2000 g for 20 min, and finally at 10,000 g for 30 min. These steps aimed to remove cellular debris, lifted cells, and large vesicles, respectively. Subsequently, the samples were ultracentrifuged at 100,000 g for 120 min at 4 °C, resulting in the pellet containing the EVs.

### Quantification of extracellular vesicles

The size and concentration of EV-iPSCs (Supplementary Fig. 1) were assessed using nanoparticle tracking analysis (NTA) with the Malvern NanoSight (NS300) System (Worcestershire, UK), which utilizes mirror light and Brownian motion to determine the size distribution and count of suspended particles. Three replicate samples (1 mL in PBS) were diluted and injected into the chamber. The vesicles were tracked and measured for 30 s, with three measurements performed for each sample at a constant flow. The video data were processed by the NanoSight NTA 3.0 software, which tracked the particles in all individual samples and utilized the Stokes–Einstein equation to calculate their hydrodynamic diameter.

### Mesangial cell culture

MMCs, obtained from ATCC (CRL-1927), were cultured in 6-well plates at 37 °C in Dulbecco's Modified Eagle’s medium/Ham's F12 medium (DMEM/F12; 3:1 mixture) supplemented with 10% fetal bovine serum (FBS), and penicillin (50 U/mL). The plates were maintained in an environment with 95% air atmosphere and 5% CO_2_. Upon reaching confluence, MMCs were subjected to a 24-h exposure to DMEM/F12 medium without FBS, according to the following experimental groups: Control group (Control), cells maintained in DMEM/F12 medium; TGF-β group (TGF-β), cells maintained in DMEM/F12 medium containing 5 ng/mL recombinant TGF-β (R&D Systems); control group with EV-iPSCs (Control + EVs), cells maintained in DMEM/F12 medium and treated with EV-iPSCs; and TGF-β treated with EV-iPSCs group (TGF-β + EVs), cells cultured in DMEM/F12 medium containing 5 ng/mL TGF-β and treated with EV-iPSCs. The concentration of TGF-β was determined before the experiment using a dose–response curve, considering its impacts on fibronectin and TGF-β gene expression. Supplementary Fig. 2 illustrates that the concentrations of 5 and 10 ng/mL had similar effects. Hence, a concentration of 5 ng/mL was chosen for the experiments. The evaluation of MMCs morphology was assessed by the phalloidin staining. Following TGF-β treatment, the MMCs showed a slight but visible change in the cell morphology, changing from a cuboidal shape to an elongated shape (Supplementary Fig. 3). The concentration of EV-iPSCs used in experiments was calculated using the number of EVs released by 10^6^ iPSCs to stimulate the same amount of MMCs (Supplementary Fig. 1). Therefore, since 10^6^ iPSC released 1.0 × 10^9^ particles/mL, we adopted this concentration to stimulate 10^6^ MMCs.

### EV-iPSCs incorporation by MMCs Assay

Initially, isolated EV-iPSCs were incubated with the fluorescent dye CellTrackerTM Red CMTPX 20 µM (Invitrogen by Thermo Fisher Scientific, USA) at a room temperature of 37 °C for 45 min. This dye contains chloromethyl or bromomethyl groups that react with thiol groups through enzymatic mediation by glutathione-S-transferase. The dye is designed to penetrate the cell membranes and transform into impermeable reaction products. Following this, the EV-iPSCs were subjected to another round of ultracentrifugation to obtain a pure pellet. MMCs were then exposed to the labeled EV-iPSCs for 1 h and visualized using an automated cell imaging system (GE Healthcare, Life Sciences, IN Cell Analyzer 2200, UK).

### mRNA expression by RT-PCR

According to the manufacturer’s instructions, the mRNA expression levels were estimated by quantitative RT-PCR. The total RNA was purified from MMCs using a commercial TRIzol (Gibco BRL, Rockland, MD, USA). The RNA quantity and purity were determined using the NanoVue spectrophotometer (GE Healthcare Life Sciences, USA). Total RNA was treated with DNase (Promega, Madison, WI, USA) to prevent genomic DNA contamination and DNase inactivation. The RNA pellet was resuspended in RNase-free water, and reverse transcripted into cDNA using a High-Capacity cDNA Reverse Transcription Kit (Applied Biosystems). RT-PCR amplification was performed using SYBR Green (Applied Biosystems) in the QuantStudio (TM) 7 Flex System (Applied Biosystems), with specific primers for each molecule as follows (sense and antisense, respectively): fibronectin (5′ acactaacgtaaattgcccca 3′ and 5′ gctaacatcactggggtgtggat 3′), vimentin (5′ aggtggatcagctcaccaatgaca 3′ and 5′ tcaaggtcaagacgtgccagagaa 3′), α-SMA (5′ tattgtgctggactctggagatgg 3′ and 5′ agtagtcacgaaggaatagccacg 3′), collagen I (5′ tgactggaagagcggagagtact 3′ and 5′ cagacgtgcttcttttccttgg 3′), collagen IV (5′ tgcaaaccctgaaaatactccac 3′ and 5′ gaggtgtatagatagccaaagccaa 3′), renin (5′ cagggagagtcaaaggtttc 3′ and 5′ gggagagaatgtggtcaaag 3′), angiotensinogen (5′ tggaagcagcagccagacac 3′ and 5′ ggcagcaagaactgggtcag 3′), AT_1_ receptor (5′ gcactcaagcctgtctacgaaaat 3′ and 5′ cctgtcactccacctcagaaca 3′), p50 (5′ atctatgatagcaaagcccc 3′ and 5′ tggatgttcatctttcttgaac 3′), p65 (5′ aggcttctgggccttatgtg 3′ and 5′ tgcttctctcgccaggaatac 3′), IKKα (5′ gtcaggaccgtgttctcaagg 3′ and 5′ gcttctttgatgttactgagggc 3′), SMAD-3 (5′ aggaatttgctgccctcctag 3′ and 5′ gcctttgacgaagctcatgc 3′), SMAD-7 (5′ caagagccctccctggatatc 3′ and 5′ tctgtaccagctgactcttgttgtc 3′), TGF-β (5′ aactattgcttcagctccacagaga 3′ and 5′ agttggatggtagcccttg 3′), and HPRT (5′ ctcatggactgattatggacaggac 3′ and 5′ gcaggtcagcaaagaacttatagcc 3′). The relative gene expression was calculated using the RT-PCR conditions under which the amplification curve was logarithmic. The mRNA expression levels were normalized to HPRT expression.

### Western blot analysis

Total protein was extracted from both MMCs and EV-iPSCs using a commercial TRIzol kit (Gibco BRL, Rockland, MD, USA) following the manufacturer’s instructions. The protein concentration was determined using the Lowry method (DC Protein Assay; Bio-Rad Laboratories Inc., Richmond, CA, USA). Equal amount of total extracted protein (20 µg) was separated by SDS–polyacrylamide gel electrophoresis (SDS-PAGE) and transferred onto nitrocellulose membranes (Amersham Pharmacia Biotech, Piscataway, NJ, USA). Immunoblot analyses were performed using primary polyclonal antibodies targeting CD9 (1:100; Abcam, USA), CD63 (1:100; Abcam, USA), CD81 (1:1000; Thermo Fisher Scientific, USA), calnexin (1:1000; Abcam, USA), vimentin (1:2000; Abcam, Brazil), α-SMA (1:2000; Sigma, USA), renin (1:1000; Novus Biologicals; USA), angiotensinogen (1:1000; Novus Biologicals; USA), p50 (1:1000; Cell Signaling, Brazil), p65 (1:2000; Merck, Brazil), IKKβ (1:1000; Cell Signaling, Brazil), and β-actin (1:20,000; Merck, Brazil). The protein expression levels were normalized to β-actin. Immunodetection involved incubation of the blots with horseradish peroxidase (HRP)-conjugated anti-rabbit secondary antibody (1:30,000, Santa Cruz Biotechnology, USA), anti-goat secondary antibody (1:7500, Santa Cruz Biotechnology, USA), or anti-mouse secondary antibody (1:30,000, Sigma, USA). Protein bands were visualized using Immobilon Western HRP substrate (Millipore, Billerica, Massachusetts, USA), and the resulting bands were quantified using Luminescent Image Alliance 4.7 (Uvitec Cambridge, UK).

### Immunofluorescence

MMCs were cultured in 96-well plates kept in the dark. Afterward, the cells were fixed with 3% formaldehyde for 15 min, followed by a wash with PBS and permeabilization with saponins for 5 min. The cells were then incubated overnight with primary antibodies against phalloidin (1:1000; Abcam, USA), fibronectin (1:250; Santa Cruz Biotechnology, USA), angiotensinogen (1:250; Novus Biologicals; Brazil), and AT_1_ receptor (1:200; Immuny Rhea Biotech, Brazil). The primary antibodies were diluted in PBS containing 3% FBS. Following a 12-h incubation, the cells were washed with PBS and exposed to fluorescent marker-conjugated secondary antibodies (FITC) for 1 h at room temperature in a dark room. Subsequently, the cells were washed with PBS containing 0.5% FBS. To stain the nucleus, the sample was treated with DAPI reagent (4′,6′-diamino-2-phenyl-indole; Invitrogen Life Technologies Inc., Gaithersburg, MD, USA) diluted in PBS for 5 min. The reagents were then removed using PBS, and the MMCs were visualized using an automated cell imaging system (GE Healthcare, Life Sciences, IN Cell Analyzer 2200, UK). Immunofluorescent staining was quantified using the same software, measuring the mean fluorescence intensity. The quantification of immunofluorescence (%) was normalized relative to the control group. Graphs were generated to display the mean fluorescence corresponding to the membranous and cytoplasmic localization of the protein in each cell.

### Statistical analysis

The results are presented as the mean $\pm$ standard error of the mean (SEM). Statistical analysis was conducted using one-way analysis of variance (ANOVA), followed by Tukey’s test. P-values below 0.05 were considered statistically significant. Graphs were generated, and statistical analysis was performed using GraphPad Prism software version 8.00 (GraphPad Software, San Diego, CA, USA).

### Supplementary Information

Supplementary Information 1.

Supplementary Figure S1.

Supplementary Figure S2.

Supplementary Figure S3.

## Data Availability

All data generated or analyzed during this study are included in this published article [and its supplementary information files].
