# Supplementary material for: Effect of extracellular vesicles derived from induced pluripotent stem cells on mesangial cells underwent a model of fibrosis in vitro
Source: Sci Rep. 2023 Sep 21;13:15749. doi: 10.1038/s41598-023-42912-6 (PMC10514265; doi:10.1038/s41598-023-42912-6)
Supplement: Supplementary file 1 — Supplementary Information 1. [file 41598_2023_42912_MOESM1_ESM.docx]

**Supplementary Figures**

**Determination of the concentration of EV-iPSCs**

The concentration of EV-iPSCs used in experiments was calculated using the number of EVs released by 10^6^ iPSCs in each amount of 10^6^ MMCs (Supplementary Figure 1).  Therefore, the concentration of EV-iPSCs added to the culture medium was 1.0 x 10^9^ particles/mL.

**Supplementary Figure 1.** **Concentration of EV-iPSCs. 10^6^** iPSCs were cultured in 6-well plates. After 24 h of incubation, the EV-iPSCs were isolated from the culture medium, and the adhered iPSCs were dissociated and counted by Countess Automated Cell Counter (Thermo Scientific).

**Determination of the concentration of TGF-β**

The concentration of TGF-β to be employed in the experiments was obtained through a dose-response curve considering the ability of TGF-β to stimulate fibronectin expression. MMCs were cultured in increasing concentrations of TGF-β (1, 5 and 10 ng/mL) for 24 hours (Supplementary Figure 1). Fibronectin and TGF-β expression were evaluated by RT-PCR. There was increased mRNA expression of fibronectin and TGF-β in the groups stimulated with 5 and 10 ng/mL of TGF-β (Supplementary Figure 1A-B). However, there is no difference between these groups. Therefore, the concentration of 5 ng/mL was selected for the following experiments.

**Supplementary Figure 2.** Determination of the concentration of TGF-β. MMCs were incubated with different concentrations of TGF-β (1, 5 and 10 ng/mL) for 24 hours. The gene expression of fibronectin (**A**) and TGF-β (**B**) was evaluated by RT-PCR. HPRT was used as the reference gene. The results are expressed as the mean ± SEM. p<0.05: * vs group control; # vs group 1 ng/mL TGF-β.

**Evaluation of MMCs morphology**

The evaluation of MMCs morphology was assessed by the phalloidin staining. Following TGF-β treatment, the MMCs showed a slight but visible change in the cell morphology, changing from a cuboidal shape to an elongated shape (Supplementary Figure 3).

**Supplementary Figure 3.** Evaluation of MMCs morphology. Cytoskeleton is stained with phalloidin (green). The nucleus is stained with DAPI (blue). Colocalization of all markers. Images are representative.

**Western Blot images:**

**CD9**

**
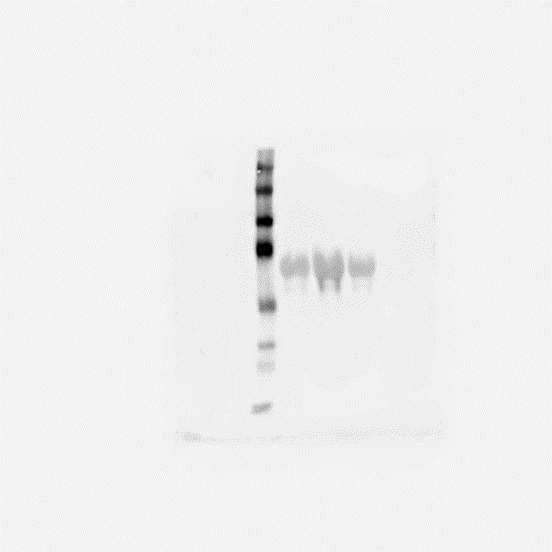

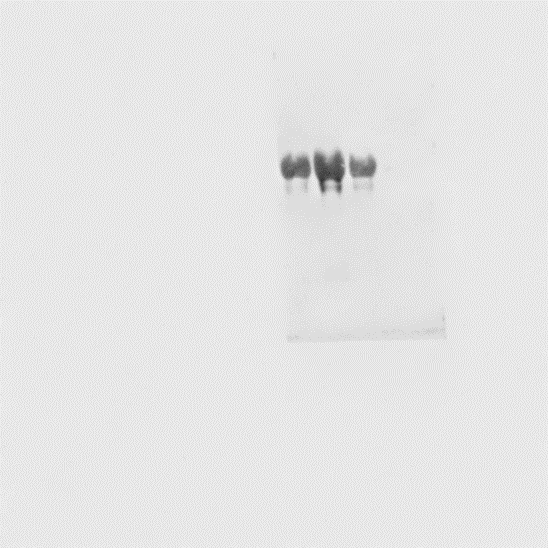
**

**CD63**

**
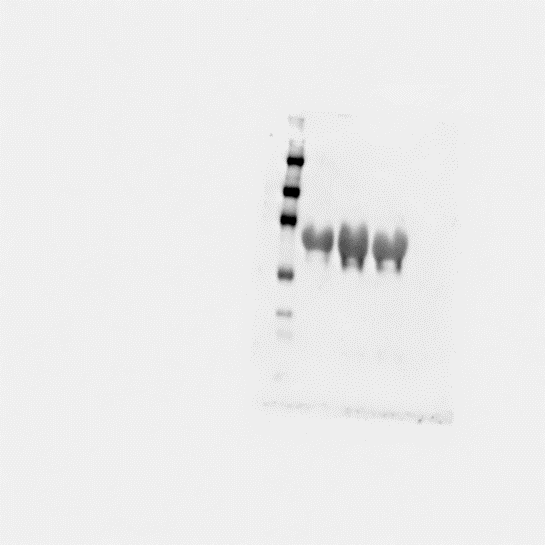

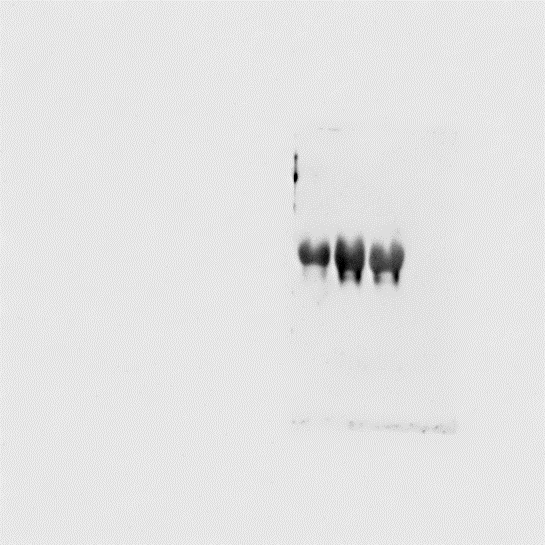
**

**CD81**

**
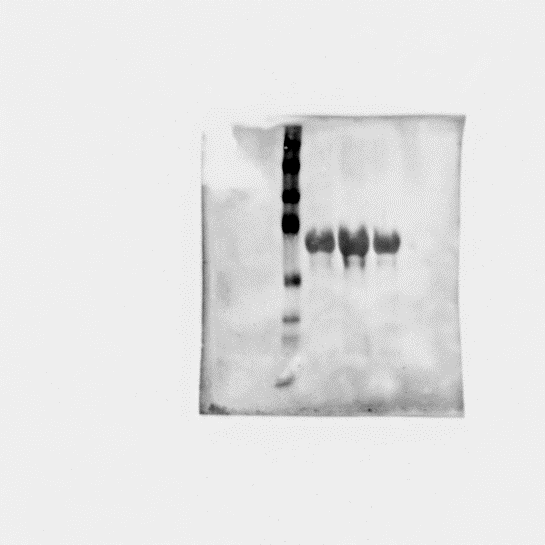
**

**Calnexin**

**
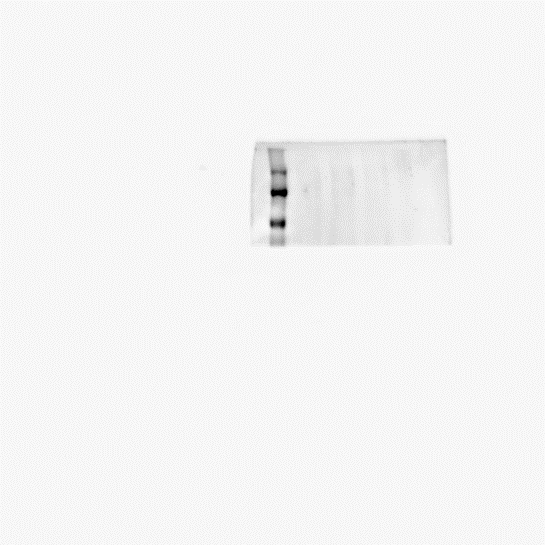
**

**Vimentin**

**
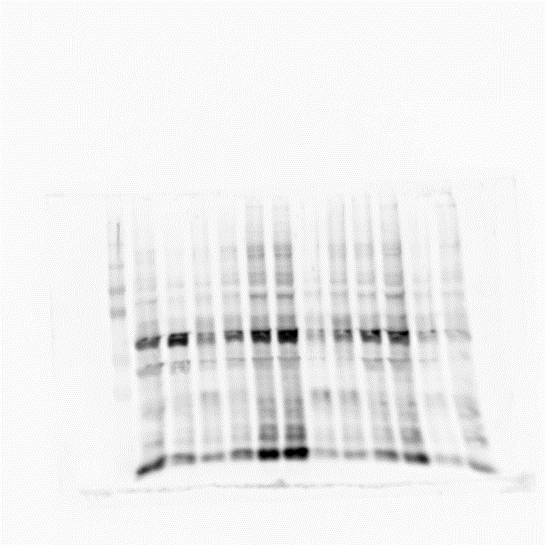
**

**α-SMA**

**
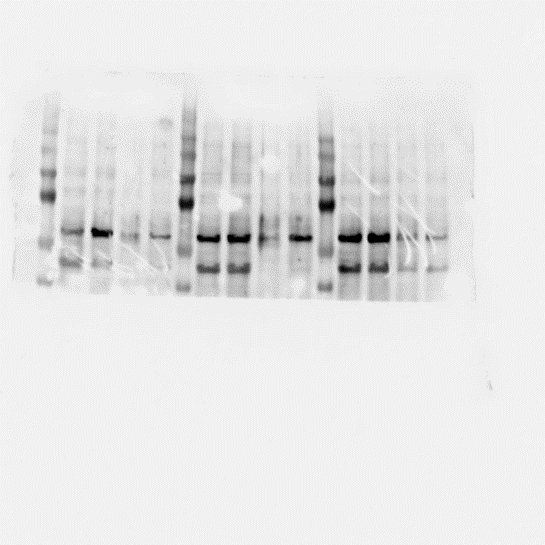
**

**β-actin**

**
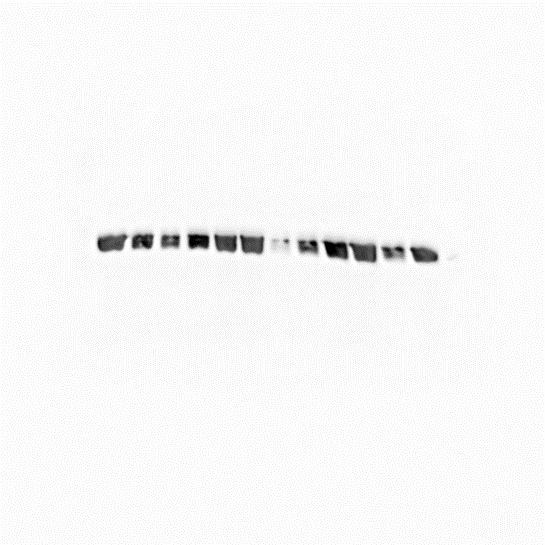

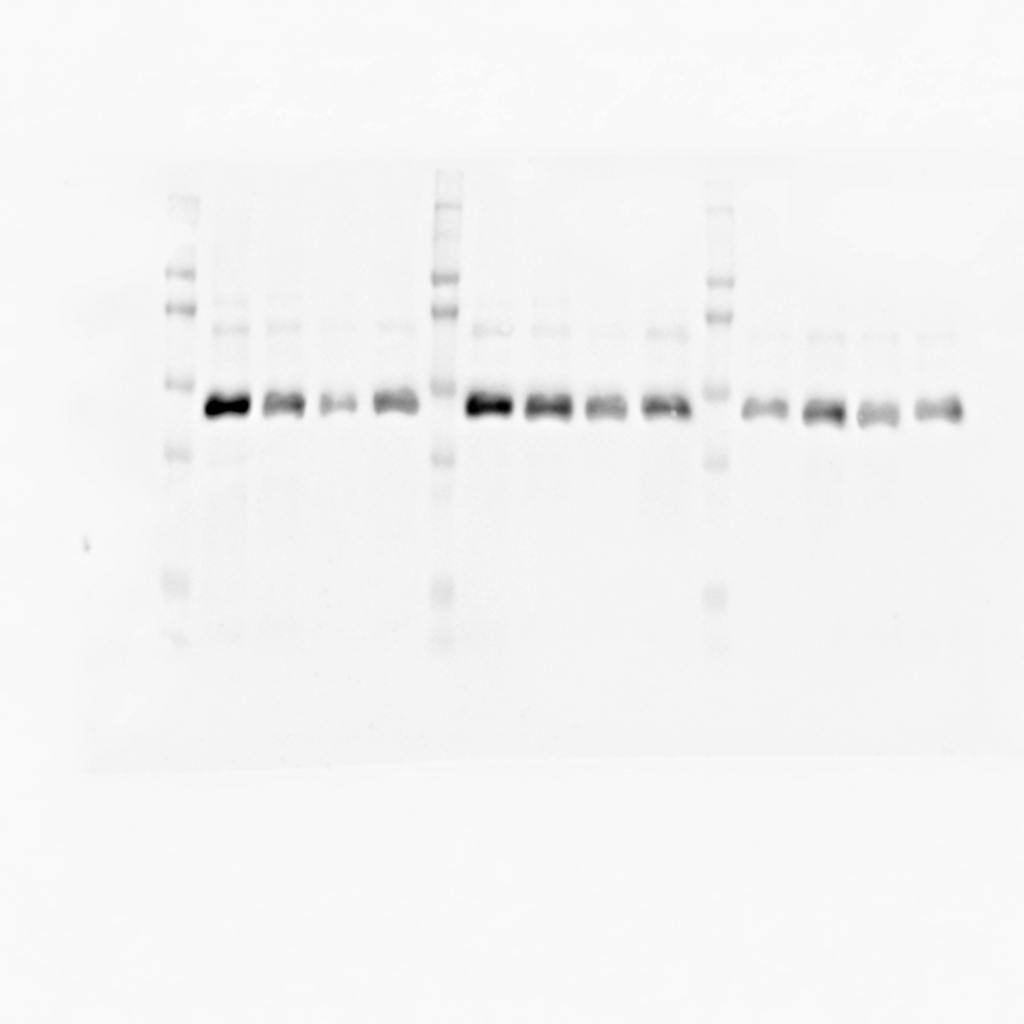
**

**
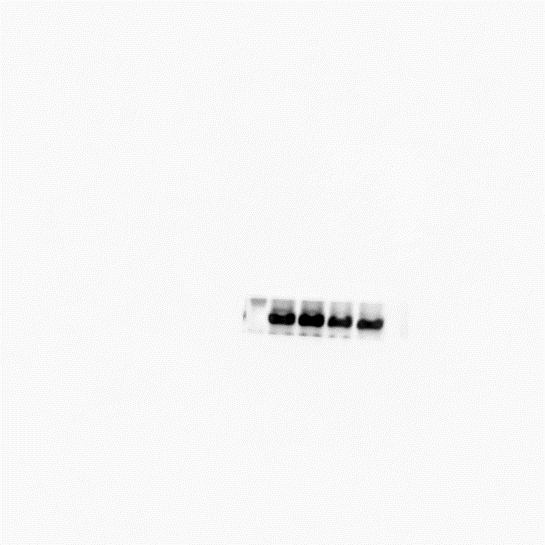
**

**Renin**

**
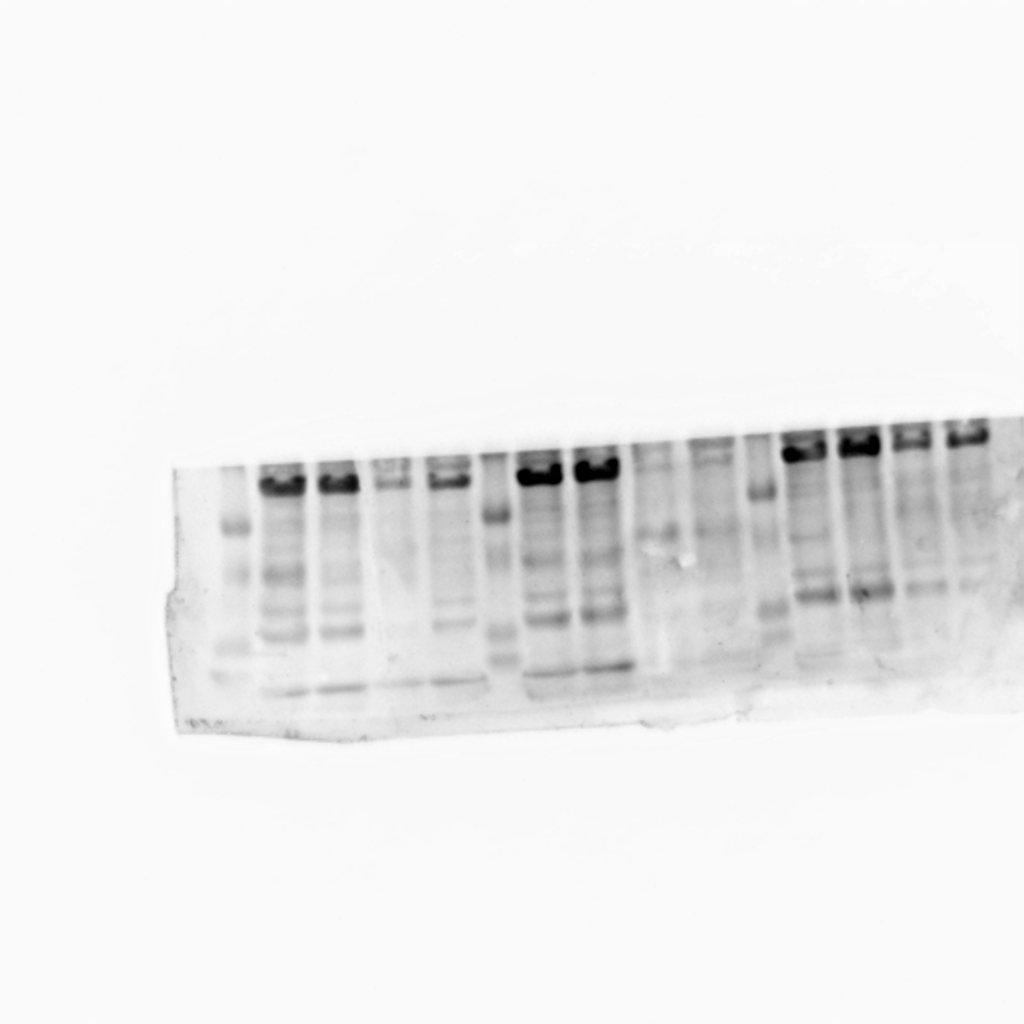
**

**Angiotensinogen**

**
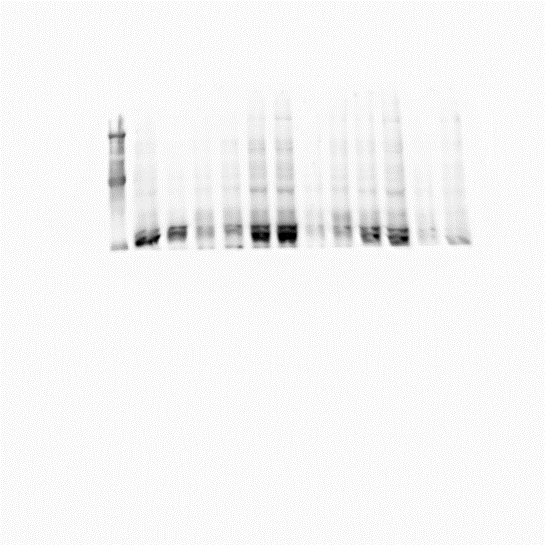
**

**p50**

**
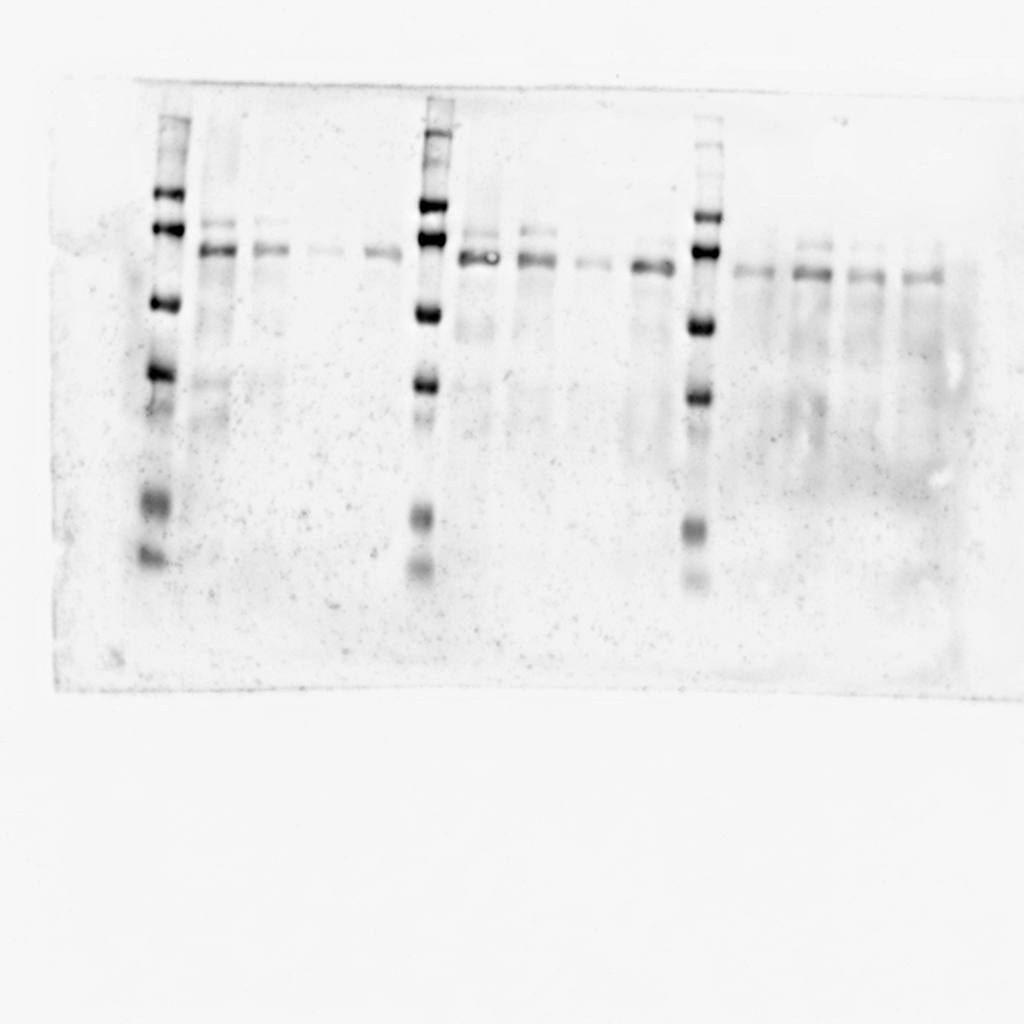
**

**p65**

**
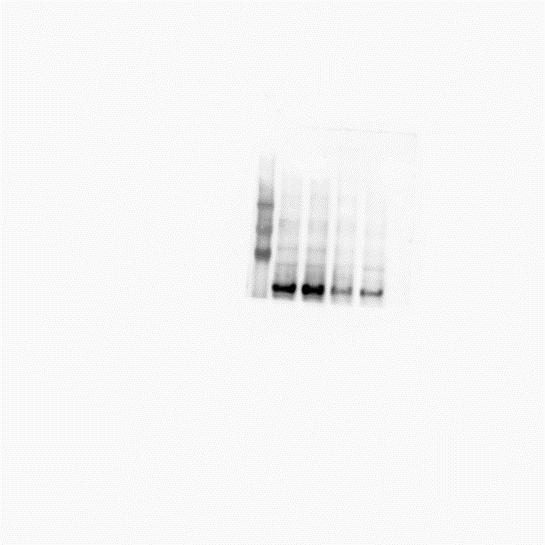
**

**IKKβ**

**
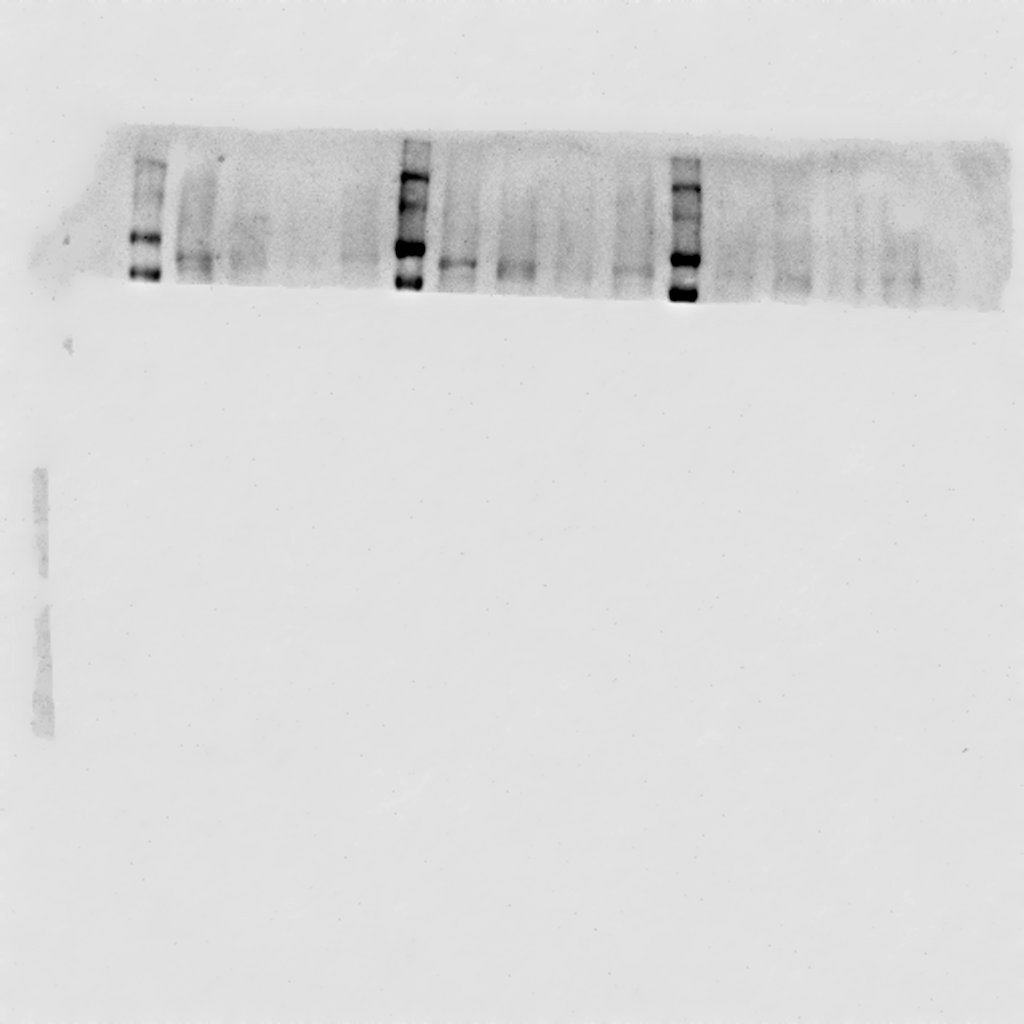
**
